# Restricting Unhealthful Food Advertising to Children and the First Amendment [Response to Letter]

**Published:** 2011-12-15

**Authors:** Jennifer L. Harris, Samantha K. Graff

**Affiliations:** Rudd Center for Food Policy and Obesity, Yale University, New Haven, Connecticut; Public Health Law & Policy, Oakland, California

## In Reply:

We agree with Dr Rossen (1) that government should be able to engage more directly in restricting advertising to children. However, the First Amendment, as currently construed by the US Supreme Court, makes content-based signage restrictions constitutionally vulnerable. During the past decade, developments in the commercial speech doctrine suggest that such restrictions would fare poorly with respect to each prong of the *Central Hudson* test.

**Prong 1.** As Dr Rossen observes, advertising to young children is inherently misleading and therefore should be subject to regulation without violating the First Amendment (1). However, the Supreme Court has repeatedly held that a child-protective speech restriction raises serious concerns if it incidentally denies adults access to information (2). In the instance of advertising on signage in the community – as opposed to, for example, during children's television or on child-directed websites – it would be difficult for the government to justify a ban because advertisers could simply assert that the messages are directed to an adult audience.

**Prong 2.** Dr Rossen makes a valid point that the government's interest in protecting public health would seem to provide a stronger rationale for restricting advertising to children than does minimizing store window clutter or preserving esthetics. But Supreme Court doctrine makes it much harder for the government to defend advertising regulations that are content-based (eg, targeted at advertisements only for certain products) as opposed to those that are content-neutral (eg, targeted at all advertisements, regardless of their messages, in a particular location) (3). Moreover, the Supreme Court indicated in a major decision in 2011 that it will look unfavorably on regulations that "discriminate" against commercial speakers on the basis of what they are saying (4).

**Prong 3.** As Dr Rossen observes, there is little evidence of the effect of "specific local policies to restrict advertising" and it is hard to collect those data, especially *before* enacting the desired change. Without such evidence, it is more difficult for the government to defend its policy. And in these cases, it is not the government that gets the benefit of the doubt but the industry (5).

**Prong 4.** We concur that local government policies restricting advertising must – in the eyes of a reviewing court – be both broad enough to be effective and simultaneously narrow enough to not restrict more speech than necessary to effect their purpose. A US Supreme Court decision handed down the year after the *Lorillard* case that Dr Rossen cites stated that "if the Government can achieve its interests in a manner that does not restrict commercial speech, or that restricts less speech, the Government must do so" (6). This is indeed a "fine line," as Dr Rossen observes. Further, it is a line likely to be drawn in industry's favor, given the similarity between the facts in *Lorillard* and a potential law restricting food advertisements on signs and billboards. In addition, the Supreme Court has in the decade since *Lorillard*, made it increasingly difficult to navigate prongs 3 and 4 of the *Central Hudson* test.

Certainly there are jurisdictions in the United States that are willing and able to enact and defend laws that push the ever-changing limits of the First Amendment. But we thought that it would be most useful for most state and local governments if we presented policies to reduce harmful food marketing to children that are likely to avoid, or at least withstand, a First Amendment challenge.
